# Antigenic Complementarity in the Origins of Autoimmunity: A General Theory Illustrated With a Case Study of Idiopathic Thrombocytopenia Purpura

**DOI:** 10.1080/17402520600578731

**Published:** 2006-03

**Authors:** Robert Root-Bernstein, Jacob Couturier

**Affiliations:** Department of Physiology Michigan State University East Lansing MI USA

## Abstract

We describe a novel, testable theory of autoimmunity, outline novel predictions made by the theory, and illustrate its application to unravelling the possible causes of idiopathic thrombocytopenia purpura (ITP). Pairs of stereochemically complementary antigens induce complementary immune responses (antibody or T-cell) that create loss of regulation and civil war within the immune system itself. Antibodies attack antibodies creating circulating immune complexes; T-cells attack T-cells creating perivascular cuffing. This immunological civil war abrogates the self-nonself distinction. If at least one of the complementary antigens mimics a self antigen, then this unregulated immune response will target host tissues as well. Data demonstrating that complementary antigens are found in some animal models of autoimmunity and may be present in various human diseases, especially ITP, are reviewed. Specific mechanisms for preventing autoimmunity or suppressing existing autoimmunity are derived from the theory, and critical tests proposed. Finally, we argue that Koch's postulates are inadequate for establishing disease causation for multiple-antigen diseases and discuss the possibility that current research has failed to elucidate the causes of human autoimmune diseases because we are using the wrong criteria.


*Clinical & Developmental Immunology*, March 2006; 13(1): 49–65

Taylor & Francis

Taylor & Francis Group

### Antigenic complementarity in the origins of autoimmunity: A general theory illustrated with a case study of idiopathic thrombocytopenia purpura

ROBERT ROOT-BERNSTEIN & JACOB COUTURIER

*Department of Physiology, Michigan State University, East Lansing, MI, USA*

**Abstract**

We describe a novel, testable theory of autoimmunity, outline novel predictions made by the theory, and illustrate its application to unravelling the possible causes of idiopathic thrombocytopenia purpura (ITP). Pairs of stereochemically complementary antigens induce complementary immune responses (antibody or T-cell) that create loss of regulation and civil war within the immune system itself. Antibodies attack antibodies creating circulating immune complexes; T-cells attack T-cells creating perivascular cuffing. This immunological civil war abrogates the self–nonself distinction. If at least one of the complementary antigens mimics a self antigen, then this unregulated immune response will target host tissues as well. Data demonstrating that complementary antigens are found in some animal models of autoimmunity and may be present in various human diseases, especially ITP, are reviewed. Specific mechanisms for preventing autoimmunity or suppressing existing autoimmunity are derived from the theory, and critical tests proposed. Finally, we argue that Koch's postulates are inadequate for establishing disease causation for multiple-antigen diseases and discuss the possibility that current research has failed to elucidate the causes of human autoimmune diseases because we are using the wrong criteria.

**Keywords:** *Circulating immune complexes, complementary antigens, idiotype–antiidiotype, ITP, Koch's postulates, theory of autoimmunity*

### Introduction

The origins of human autoimmune diseases remain one of the outstanding mysteries of modern medicine. Standard textbook accounts attribute induction of autoimmunity to one of five, non-exclusive, processes: (1) molecular mimicry between foreign antigens and "self" determinants that results in cross-reactivity of idiotypic antibodies with the "self" determinants; (2) induction of anti-idiotypic antibodies that cross-react with "self" determinants following a normal idiotypic response to a foreign antigen; (3) release of sequestered or "hidden" alloantigens that activate an active immune response; (4) incomplete clonal deletion, which leaves auto-reactive T- or B-cell clones available for activation by foreign antigens; (5) genetic predisposition to autoimmunity. While some evidence exists to support each of these processes, no coherent theory of autoimmunity that integrates all available observations exists, nor have any of the current theories led to the development of autoimmune disease models in animals that reproduce naturally occurring processes of autoimmunity induction. This paper attempts to provide an integrated, testable theory of autoimmune disease induction that provides strategies for identifying infectious agents involved in disease induction. We demonstrate the manner in which the theory can be applied and tested with regard to idiopathic thrombocytopenia purpura (ITP).

### Theory

The theory presented here posits a process that can break "self" tolerance by confusing the immune system itself. The process involves provoking the immune system with a pair of molecularly complementary antigens (at least one of which mimics a

Correspondence: R. Root-Bernstein, Department of Physiology, 2174 Biomedical and Physical Sciences Building, Michigan State University, East Lansing, MI, USA. Tel: 1 517 355 6475, ext. 1101. Fax: 1 517 355 5125. E-mail: rootbern@msu.edu

ISSN 1740-2522 print/ISSN 1740-2530 online © 2006 Taylor & Francis
DOI: 10.1080/17402520600578731

"self" determinant) that give rise to a pair of complementary immune responses that attack each other as well as a tissue or organ within the body (Figure 1). The theory asserts that pairs of complementary antigens will be processed differently than individual or non-complementary sets of antigens. (Westall and Root-Bernstein 1983a,b, 1986, Root-Bernstein and Westall 1986, Root-Bernstein 1991a,b)

Consider the immunological processing of individual antigens first. In a typical immune response, each antigen elicits a complementary antibody or T-cell response. These antibody or T-cell responses are independently controlled and non-interactive. Antigens that too closely mimic "self" determinants will not induce an immune response. The immune system down-regulates its activity against each antigen as that antigen is eliminated from the body.

Complementary antigens will be processed quite differently than individual antigens or sets of non-complemenatry antigens. Complementary antigens will form a molecular complex that is antigenically unique. While each component of such a molecular complex may mimic a "self" determinant, the complex itself will have a unique structure that does not. Thus, the complex will be antigenic even if the components individually would be too similar to "self" determinants to be antigenic. Subsequent processing of the complex results in a range of antibodies or T-cell reactive clones, some primarily against the antigenic complex, some biased towards components of the complex. It is the immunological response against self-mimicking components of the complex that result in autoimmune processes.

The results of simultaneously immunologically processing a pair of complementary antigens will result initially in abrogation of the self–nonself distinction within the immune system itself (Figure 1). To begin with, immunological response to one antigen will result in antibodies (or T-cells) complementary to that first antigen, which will mimic stereochemically the second, complementary antigen. Similarly, the immunological response to the second antigen will result in antibodies (or T-cells) complementary to the second antigen, which will mimic stereochemically the first, complementary antigen. Thus, each immunological response will mimic one of the antigens. Since the antigens are complementary, the immunological responses will also be complementary so that the resulting antibodies (or T-cells) will attack each other. Thus, while each antibody (or T-cell) response is "self", each response will be viewed by the immune system as also being "non-self". Immunological tolerance will thereby be broken, and each immune response will continuously provoke its complementary response in an unending cycle of immunological civil war.

At heart, then, this theory proposes that all autoimmune diseases begin as autoimmune responses within and against the immune system itself. Intra-immunological warfare can spread to other tissues or organs if at least one of the pair of complementary antigens mimics a "self" determinant. In these circumstances, the "self" determinant will also become a target of the now unregulated immune response and provide an essentially continuous stimulus to this response.

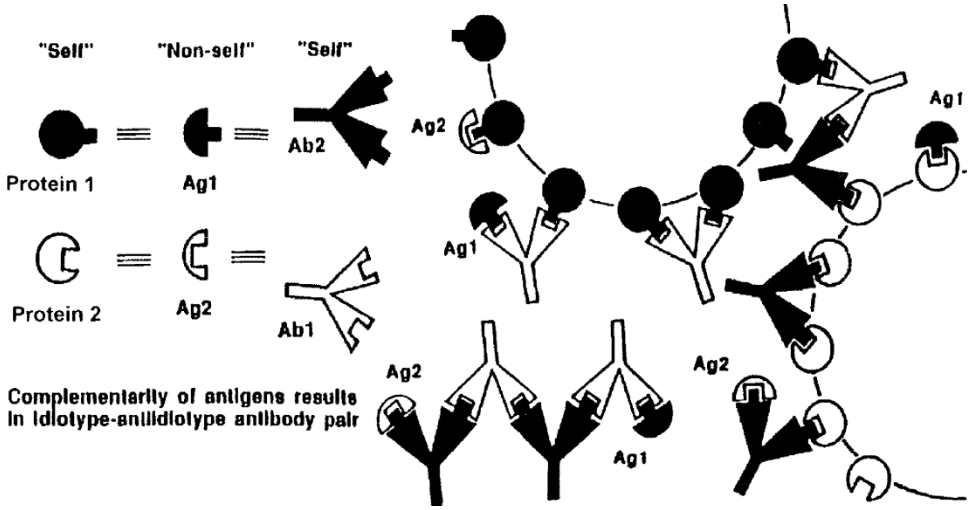

Figure 1. Schematic model of the complementary antigen theory of autoimmunity. If a pair of antigens are molecularly or stereochemically complementary (as defined by their ability to bind to each other specifically) then they will induce molecularly complementary antibody (or T-cell) responses), i.e. having an idiotype–antiidiotype relationship—but both antibodies will be idiotypic. These complementary antibodies will bind to each other and their respective antigens to create circulating immune complexes. Each antibody will also treat the other as "nonself" and therefore the self–nonself distinction will be abrogated and an immunological civil war initiated. If one or both of the antigens are molecular mimics of a self determinant, then this unregulated immunological civil war will spread to attack host tissues or organs. The same basic mechanism will occur in T-cell mediated autoimmune diseases as well.

This theory is compatible with, but not dependent upon, Jerne's network theory of immunological regulation (Steinberg and Lefkovits 1981). One can reinterpret the description given above in terms of idiotypes and antiidiotypes. A complementary pair of antigens will induce a pair of idiotypic immunological responses that behave as if they were idiotypic–antiidiotypic pairs. If one assumes that regulation of immunological responses requires a cascade of idiotype–antiidiotype responses, as Jerne's theory does, then inducing a pair of simultaneous primary idiotypic immunological responses that act as anti-idiotypic responses for each other destroys network regulation. In fact, antiidiotypic immune responses have been characterized in a wide range of auto-immune diseases including ITP, diabetes and AIDS (e.g. Shoelson et al. 1986, Balint and Jones 1994, Silvestris et al. 1994, Krook et al. 1996, Nardi and Karpatkin 2000). It is worth bearing in mind that these immune responses may not, in fact, be antiidiotypic, but primary idiotypic responses that result from a pair of complementary antigens. More on this point in the next section.

### Predictions made by the theory

The antigenic complementarity theory of autoimmunity makes testable predictions. One is that complexes of complementary antigens should be able to induce experimental autoimmune diseases. Two cases suggest that this is correct.

It has been demonstrated in studies of experimental allergic encephalomyelitis (EAE) that the minimal components necessary to induce the disease in guiniea pigs are a combination of the tryptophan peptide of myelin basic protein (EAE peptide) and muramyl dipeptide (MDP), a fragment of bacterial cell walls (Westall and Root-Bernstein 1983a,b, 1986, Root-Bernstein 1991b). Westall and Root-Bernstein have demonstrated using nuclear magnetic resonance spectroscopy and other techniques that EAE peptide has a binding site for MDP and that the two molecules bind to each other to form a stable complex (Root-Bernstein and Westall 1983, 1990, Takeuchi et al. 1990). It has also been demonstrated that insulin binds to glucagon to form a stable complex that is hyperantigenic (Root-Bernstein and Dobbelstein 2001). Moreover, all antibodies supposedly induced by insulin, whether in experimental animals or human patients with diabetes, have significantly higher affinity for an insulin–glucagon complex than for insulin alone (Root-Bernstein and Dobbelstein 2001).

These data suggest that in experimental auto-immune diseases such as autoimmune thyroiditis that are currently induced using a combination of a self-mimicking protein "antigen" (thyroglobulin) and a complex bacterial "adjuvant" (lipopolysaccharide or Freund's complete adjuvant) (e.g. Esquivil et al. 1977), a pair of complementary, binding antigens will be isolatable as the minimal necessary inducers of autoimmunity. In cases involving the use of complex antigen mixtures for the induction of autoimmune models, such as that of mycobacterial-induced adjuvant arthritis, the complementary antigen theory predicts that the minimal components will consist of a pair of antigens from the mixture. This prediction is given some credibility from the fact that no single chemical component of mycobacteria has yet been found to be able to induce adjuvant arthritis by itself and purified components (e.g. heat shock proteins and collagens) can be used to vaccinate against adjuvant arthritis (Billingham et al. 1990, Zhang et al. 1990, Yang et al. 1992, Prakken et al. 1997, Ulmansky et al. 2002). The current theory proposes that a minimum of two mycobacterial antigens having a complementary relationship will be found to be necessary to induce adjuvant arthritis.

A second major prediction made by the complementary antigen theory is that circulating immune complexes and perivascular cuffs or other immunological aggregates are results of complementary immune responses. Circulating immune complexes are highly associated with autoimmune disease (Clancy et al. 1980, Trent et al. 1980, Puram et al. 1984, Morrow et al. 1986, Kurata et al. 1987, Reddy and Grieco 1990, Stanojevic et al. 1996). In antibody-mediated forms of autoimmunity, CIC will be found to be composed not only of antibody–antigen aggregates, as standard research and textbook descriptions maintain, but also of antibody–antibody aggregates (Figure 1). Similarly, perivascular cuffs or other lymphocyte aggregates in cell-mediates auto-immune diseases will be found to be composed of complementary sets of lymphocytes that are able to bind to, and attack, each other (Root-Bernstein 1991b).

A third prediction of the theory is that post-infectious and post-vaccinal autoimmune diseases should occur most frequently in individuals who have specific pairs of concurrent, combined infections (or vaccinations superimposed upon an appropriate active infection), in which complementary antigens are present. If the theory is correct, then the more chronic, concurrent infections an individual develops, or the larger the number of distinct antigens encountered simultaneously (as in multiple vaccination programs for soldiers going overseas) the greater will be their probability of contracting a pair of complementary antigens that mimic one or more "self" determinants. People with AIDS are prototypes for this scenario and, indeed, they develop all forms of autoimmunity at hundreds of times the rate found in the general population (Morrow et al. 1991, Zandman-Goddard and Shoenfeld 2002). This fact suggests that studying the specific sets of infections that occur simultaneously in individual patients in terms of their subsequent development of autoimmune diseases may provide crucial clues to the origins of these diseases (Root-Bernstein and Hobbs 1992, Root-Bernstein and DeWitt 1994; Root-Bernstein 1995). It is important to emphasize that the theory does not predict that *any* random set of concurrent infections increases risk of autoimmunity, but only that concurrent, *complementary* infections increase the risk of autoimmunity.

Again, some existing data suggest that this prediction may be correct. For example, human immunodeficiency virus is know to bind to CD4-like regions on sperm resulting in simultaneous immunological processing of HIV and sperm antigens (reviewed in Root-Bernstein and Hobbs 1993, Root-Bernstein and Dewitt 1995). The result is induction of lymphocytotoxic autoantibodies that cross-react with sperm (Sonnabend 1989; reviewed in Root-Bernstein and Dewitt 1995). Similarly, in a study of people with AIDS, only those with active cytomegalovirus infection combined with active mycobacterial infection developed demyelinating autoimmunity (Root-Bernstein 1991a). No other infection, or group of infections, correlated with demyelinating autoimmunity. This correlation between demyelinating autoimmunity and combined CMV-mycobacterial infections is also observed in people with transplants and those on other forms of immunosuppressive therapies. Notably, CMV is known to have significant homologies with myelin basic protein, and mycobacteria are, of course, a component of Freund's complete adjuvant, suggesting that AIDS-associated demyelinization may truly be a form of human EAE. This possibility is testable by combining myelin-like CMV antigens with mycobacterial antigens to determine if a demyelinating animal model can be created.

The theory also makes a highly unusual prediction that either antigen alone can be used to vaccinate against the particular autoimmune disease that it can induce in the presence of its complement (Figure 2). This fact has been amply demonstrated in animal models such as EAE in which either myelin basic protein or mycobacterial adjuvant inoculated several days to weeks prior to their mixture prevents EAE induction (reviewed in Westall and Root-Bernstein 1983a,b, 1986, Root-Bernstein 1991b). Two, non-exclusive explanations exist for such prevention based on the complementary antigen theory. One explanation is that by introducing one antigen, the complementary antibody or T-cell clone is activated and it proliferates. Since this antibody or T-cell clone is, de facto, "self", and since it is characterized by being stereochemically complementary to the antigen, this "self" determinant is the stereochemical complement to the antigen. Thus, when a mixture of the antigen with its complement stimulates the immune system, instead of provoking an autoimmune response, the immune system will recognize the complementary antigen as being "self" and fail to produce an immune response to it. As with the general theory, this specific explanation is also compatible with Jerne's network theory of immune system regulation.

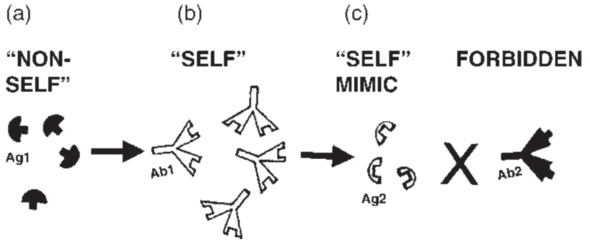

Figure 2. Schematic model of prevention according to the complementary antigen theory of autoimmunity. If a host processes one (a) of a pair of complementary antigens significantly prior to the other, then the initial antigen induces its complementary antibody (or T-cell response) first (b). When the second, complementary antigen is introduced to the immune system (c), it mimics the antibody (or T-cell) response already in place within the immune system. This existing immune response either prevents the formation of the an immune response to the complementary antigen because this antigen is recognized as "self", or, if an immune response is triggered, it will be eliminated by the pre-existing, initial antibody (or T-cell).

At the same time, another process may also be at work that prevents induction of autoimmunity following vaccination with a single antigen (Figure 2) (reviewed in Westall and Root-Bernstein 1983a,b, 1986, Root-Bernstein 1991b). By the time the complementary mixture stimulates the immune system, the immunological response to the vaccinating antigen will already be in place, or have a good head start on the complementary response. Recall that according to the complementary antigen theory, the immunological response to the complementary antigen (that is to say, the antibody or T-cells induced by the complementary antigen) will mimic the vaccinating antigen. When the complementary antigen is introduced into the immune system, any immune response (antibody or T-cell) provoked by this complementary antigen will be attacked by a more mature, pre-existing set of antibodies or T-cells. Sheer numbers dictate that the established or pre-existing immune response will eliminate any complementary immune response induced later. Again, this explanation is consistent with Jerne's network theory.

The suppression of an already-existing autoimmune disease by one of the antigens used to induce that disease can be explained by a similar process (Figure 3). Again, such suppression has been demonstrated for several animal models of autoimmunity (reviewed in Westall and Root-Bernstein 1983a,b, 1986, Root-Bernstein 1991b, see also Root-Bernstein et al. 1986). In EAE, for e.g., myelin basic protein can suppress the disease and so can mycobacterial antigens. Assuming that autoimmunity will only exist if there is a relative balance or dynamic

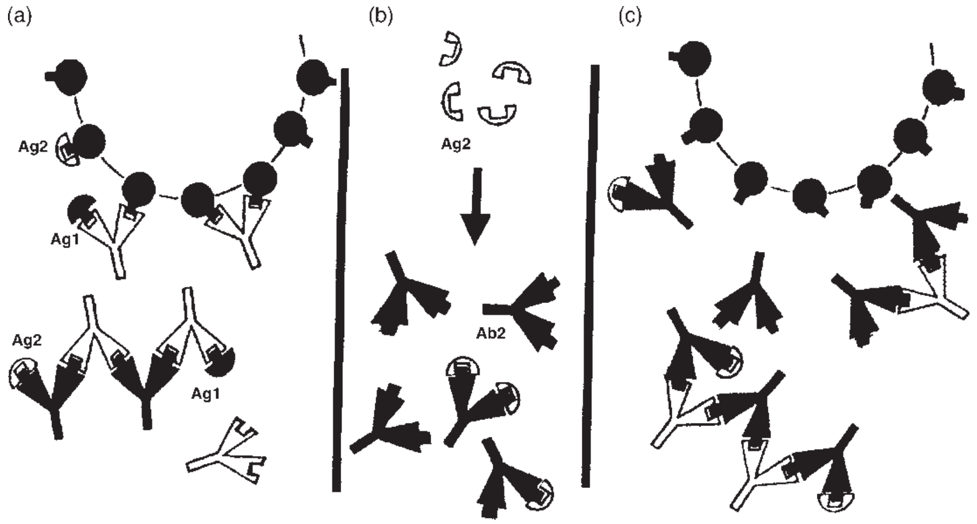

Figure 3. Schematic model of suppression of autoimmunity according to the complementary theory of autoimmunity. If autoimmunity is induced by a pair of complementary antigens as in Figure 1 (a), and a large amount of one of the antigens is then presented to the immune system (b), an increase in the immune response (antibody or T-cell) to that dose of antigen will occur. As a result of increasing one of the complementary pair of immune responses at the expense of the other (c), the autoimmunity caused by the complementary immune response will be blocked, and the enhanced immune response will eliminate the unenhanced one eliminating the autoimmune process and returning the system to normal. Note that the enhanced immune response will remain, but since normal regulation will be restored (the abrogation of the self–nonself distinction within the warring immune system eliminated), there will be no autoimmunity caused by the remaining antibody.

equilibrium between the immunological responses to the complementary antigens, it follows that anything that preferentially decreases one immune response, or preferentially increases one response, will destroy the balance, tipping the civil war in favor of one immunological response or the other. Inoculating large amounts of one causative agent into an organism with a complementary antigen-induced disease will induce a larger immune response to that antigen, allowing the immunological response to it to overwhelm the complementary immune response. Once a single immune response dominates the regulatory system, then the "self–nonself" distinction becomes unambiguous again and the autoimmune process ceases. For example, large amounts of myelin basic protein, the so-called "encephalitogen" not only suppress pre-existing EAE, but leave behind a very robust anti-myelin immune response that no longer attacks nerves. The current theory predicts that in this case, the mycobacterial response will have disappeared. Conversely, when mycobacterial antigens are used to suppress EAE, the theory predicts that anti-mycobacterial immunity will remain strong but the response to myelin basic protein will disappear. Similarly, if lipopolysaccharide is used to suppress autoimmune thyroiditis, the LPS response should remain strong following disease suppression, whereas the thyroglobulin response should disappear. And in adjuvant arthritis, heat shock protein suppression of the disease should leave a strong HSP response while eliminating the anti-collagen response.

Finally, the complementary antigen theory predicts that the time-course of an autoimmune disease will depend on three primary factors: (1) the relative balance of immune responses provoked against the pair of complementary antigens; (2) the relative accessibility and concentrations of "self" antigens that mimic the inducing antigens; and (3) the timing of exposure to the complementary antigens. If the mixture of complementary antigens is well-balanced and provokes a well-balanced set of complementary immune responses, and if each complementary antigen mimics a "self" determinant that is readily accessible to the immune system and present in reasonably high concentrations, then one would expect the resulting autoimmunity to be robust and chronic. If, however, the mixture of antigens provokes an unbalanced immune response, then one immune response will eventually eliminate the complementary one and the response will be acute. But even when a well-balanced immune response is provoked by complementary antigens, if only one of the antigens mimics a "self" determinant, or only one of a pair of mimics is readily accessible to the immune system, then the autoimmune disease will be self-limiting as one side of the immunological civil war slowly gains dominance over the other. Thus, the theory explains how varying the proportions of complementary antigens and their respective "self" mimics can modify the autoimmune disease process in ways that are seen in both animal models and human cases. Finally, the theory predicts that the antigens must be presented to the immune system simultaneously, or nearly so, in order to induce autoimmunity. If the antigens are presented at significantly different times, then autoimmunity will be prevented by the mechanisms described above.

### Case study: Idiopathic thrombocytopenia purpura

The real challenge to autoimmunity studies is, of course, to elucidate the causes of autoimmunity in human beings so that animal models can be set up that mimic natural disease progression. This goal has so far eluded investigators. Save, perhaps, for adjuvant arthritis, which actually occurs in human patients treated for cancer chemotherapy with mycobacterial adjuvants, animal models of autoimmune diseases are blatantly artificial, almost universally employing non-infectious antigens such as vertebrate hormones or proteins that are very unlikely to be involved in the actual induction of human forms of disease (reviewed in Cohen and Miller 1994; see also Oyaizu et al. 1988, Musaji et al. 2004). The complementary antigen theory of autoimmunity provides a novel strategy for elucidating natural causes of human autoimmunity.

In some types of autoimmunity, the molecular targets are well-enough defined to provide strong clues about the molecular complementarity that may be involved. One such disease is the autoimmune blood coagulation disease ITP, in which the primary molecular targets of autoantibodies are known to be platetelet glycoprotein 1b (pgp 1b) and von Willebrand's factor (VWF, or factor IX) (Kahane et al. 1981, He et al. 1994, 1995, Hou et al. 1997, Wadenvik et al. 1998, Stéphan et al. 2000, McMillan 2003). Notably, pgp 1b binds to VWF during the normal course of the blood coagulation cascade, and the binding regions of each molecule for the other have been reasonably well characterized: the A1 binding domain of the mature VWF glycoprotein ($Gly^{479}$ to $Pro^{717}$) interacts with the Platelet gp Ib Platelet gp Ib α-chain ($His^{1}$–$Arg^{293}$) (Titani et al. 1987, Vicente et al. 1988, Emsley et al. 1998, Cruz et al. 2000, Shimizu et al. 2004).

Since the molecular targets of ITP are known, and these targets are molecularly complementary, it is possible to use homology searching to identify sets of potentially complementary antigens within possible infectious agents that might be causative agents. The search for such complementary antigens is greatly facilitated by the fact that epidemiological studies have identified a narrow range of infectious agents that are highly associated with onset of ITP. These include HIV-1, the herpes viruses, including cytomegalovirus, *Streptococcus*, *Mycobacterium*, rubella virus, varicella virus and *Helicobacter* as some of the most documented infectious agents linked to ITP (Kahane et al. 1981, Van Spronsen and Breed 1996, Wright et al. 1996, Bar Meir et al. 2000, Humblot et al. 2001, Candelli et al. 2003, Fisgin et al. 2003, Ichiche et al. 2003, Takahashi et al. 2004). Notably, many reports document concurrent infections with two or more of these agents (e.g. Rahal et al. 1968, Hamner et al. 1996, Kouwabunpat et al. 1999, Sakata et al. 1999).

Using these molecular and epidemiological clues in concert, we set out to determine whether two of the most common agents associated with ITP onset—cytomegalovirus (CMV) and streptococcus (specifically group A) contained antigens with significant homologies to the complementary binding regions of pgp1b and VWF. We also tested antibodies against pgp1b and VWF to see: (1) whether they were complementary to each other, as would be predicted by the molecular complementarity of their antigens; (2) whether any of the antibodies against the infectious agents identified epidemiologically as related to ITP were complementary to each other; (3) whether any of the antibodies against infectious agents were complementary to pgp1b or VWF. The object of these studies was to determine whether it was possible to identify a set of complementary antigens that could give rise to a set of complementary antibodies that have specificity for the targets of ITP in human beings, pgp1b and VWF. There is one further step to this process that we have not yet attempted, which is the use of antigens identified by this process to induce a novel animal model of ITP for the purposes of studying methods for prevention and treatment of ITP.

### Materials and methods

### *Homology searches*

Homology searches were conducted between various GAS proteins and both VWF (Swiss-Prot ID: P04275) and Platelet gp Ibα (Swiss-Prot ID: P07359), and between various CMV proteins and both VWF and Platelet GP Ibα. Pearson's LALIGN program from the FASTA sequence alignment analysis program was employed for the homology scans at the following EMBnet organization URL: http://www.ch.embnet.org/software/LALIGN_form.html (Pearson and Lipman 1988, Huang and Miller 1991). The following alignment parameters were selected: "local" was selected for "Alignment method"; "Scoring matrix" was left at "default"; "Opening gap penalty" was set at " − 14"; and "Extending gap penalty" was set at " − 4". The significance of each homology was determined based on a numerical scoring method as previously described (Root-Bernstein and Hobbs 1992; 1993, Root-Bernstein and Dobbelstein 2001, Root-Bernstein 2004, Root-Bernstein and Rallo 2004, Root-Bernstein 2005a,b): briefly, an identical amino acid was assigned a score of one; conservative substitutions were assigned a score of one-half; and a homology was considered to be significant if a score of five or greater (i.e. at least 50% homology within ten consecutive amino acids) was attained. This degree of homology is also considered to be significant by other laboratories (31–34). For the hypothesis under consideration, special focus was directed towards the alignments that yielded homologies within the VWF A1 binding domain of the mature glycoprotein ($Gly^{479}$–$Pro^{717}$) for Platelet gp Ibα, and within the Platelet gp Ibα binding domain ($His^{1}$–$Arg^{293}$) for VWF (35–41) since these constitute the complementary binding regions of the two proteins.

### *Double-antibody (DA) ELISA*

85 different combinations between viral–bacterial, viral–host protein, and bacterial–host protein antibodies were tested for complementarity using DA-ELISA, a simple modification of the standard direct and indirect ELISAs in which an antibody is adsorbed onto the microplate well instead of antigen (Root-Bernstein 1995, Root-Bernstein and Dobbelstein 2001, Root-Bernstein and Rallo 2004, Root-Bernstein 2004, Root-Bernstein 2005a,b). The strength of binding between the two antibodies can be determined by serially diluting the antibody to be adsorbed onto the solid phase, and then adding a second antibody (preferably enzyme-conjugated) at a constant concentration. If the second antibody is not enzyme-conjugated, then an appropriate enzyme-conjugated anti-IgG that is specific for the second antibody is then added. The 21 viral, bacterial, and human antibodies tested for complementarities are specified in Table I.

Briefly, serially diluted (PBS pH 7.4) antibody (1 mg/ml) to be adsorbed onto the microplate well was added (100 μl/well) to round-bottom 96-well microplates (Costar) and incubated for at least 2 h with agitation at room temperature. Following adsorption, wells were washed with a manual plate washer (Biotrak) with 0.1% Tween-20 solution. Wells were then blocked with saturated polyvinyl alcohol (PVA) solution (200 μl/well) and incubated for 1 h. Following blocking and washing, second antibody was added (100 μl/well) and incubated for 1 h. If necessary, appropriate anti-IgG was then added (100 μl/well) and incubated for 1 h. Following incubation and washing, ABTS (Chemicon International, Inc-Temecula, CA) was then added (100 μl/well) for color development and the reaction stopped after 20–30 min. All washings were done 3× and all experiments were performed in triplicate. Absorbances were read at 405 nm with a SpectraMax 340 spectrophotometer (Molecular Devices-Sunnyvale, CA). Non-specific binding of second antibody was determined by coating wells with PVA only and then subtracting this absorbance from the absorbances of experimental wells. Data was analyzed with Softmax™ Pro and graphed with Excel.

### *Double-(monoclonal) antibody ELISA*

Based on the DA-ELISA results that indicated complementarity between certain polyclonal antibodies (PABs), we further modified the DA-ELISA described above in an effort to determine if the complementarities could be identified to the level of monoclonal specificity between MAB × Platelet gp Ib and the various CMV MABs (IEA, EA, SLA, gpB, gp gH, 65 kDa). Since MABs are not manufactured in enzyme-conjugated form, such an ELISA derivation would involve adsorption of one MAB onto the solid phase, blockage with PVA, addition of the second MAB (to be tested for its complementarity to the first MAB), and finally addition of anti-Ms IgG-HRP. However, this procedure would yield misleading results since one would not be able to discern how much of the binding activity is attributable to binding between the first and second MAB, to binding between the first MAB and anti-Ms IgG-HRP, or to binding between the second MAB and anti-Ms IgG-HRP. To determine if these distinctions could be achieved, three different binding assays (ie. three different assays for each MAB-to-MAB combination) were conducted simultaneously (preferably all on a single 96-well microplate). To our knowledge, such an ELISA derivation has not been reported by other investigators.

Assay 1: serially diluted (PBS pH 7.4) MAB × CMV (1 mg/ml) to be adsorbed onto the microplate well was added (100 μl/well) to round-bottom 96-well microplates (Costar) and incubated for at least 2 h with agitation at room temperature. Following adsorption, wells were washed with a manual plate washer (Biotrak) with 0.1% Tween-20 solution. Wells were then blocked with saturated PVA solution (200 μl/well) and incubated for 1 h. Following blocking and washing, MAB × Platelet gp Ib at constant concentration was added (100 μl/well) and incubated for 1 h. Following incubation and washing, anti-Ms IgG-HRP was then added (100 μl/well) and incubated for 1 h.

Assay 2 (assessment of binding between MAB × CMV and anti-Ms IgG-HRP): serially diluted (PBS pH 7.4) MAB × CMV (1 mg/ml at the same dilution as in Assay 1) to be adsorbed onto the microplate well was added (100 μl/well) to round-bottom 96-well microplates (Costar) and incubated for at least 2 h with agitation at room temperature. Following adsorption, wells were washed with a manual plate washer (Biotrak) with 0.1% Tween-20 solution. Wells were then blocked with saturated PVA solution (200 μl/well) and incubated for 1 h. Following blocking and washing, anti-Ms IgG-HRP at the same

Table I. Platelet glycoprotein 1b sequence homologies with group A streptococcus proteins. The glycoprotein region used for homology searching is that associated with von Willebrand factor binding (see text).

concentration as in Assay 1 was then added (100 μl/well) and incubated for 1 h.

Following incubation and washing after addition of anti-Ms IgG-HRP to all assays, ABTS (Chemicon) was added for color development to all three assays simultaneously, and absorbances were also subsequently read for all three assays simultaneously. All washings were done 3 × and all assays were performed in triplicate. Absorbances were read at 405 nm with a SpectraMax 340 spectrophotometer (Molecular Devices). Non-specific binding was determined by coating wells with PVA only and then subtracting this absorbance from the absorbances of experimental wells. Data was analyzed with Softmax™ Pro and graphed with Excel.

### Results

Homology searching demonstrates that both GAS and CMV have multiple, statistically significant, sequences homologous with both regions of VWF and pgp1b that are associated with the binding of VWF to pgp1b (Tables I–IV). Such numerous and significant CMV and GAS homologous regions are not found to other human proteins such as the insulin receptor and glucagon receptor (data not shown) or

Table II. Von Willebrand factor (factor IX) sequence homologies with cytomegalovirus proteins. The von Willebrand factor region used for homology searching is that associated with platelet glycoprotein binding (see text).

between VWF and pgp1b and other viruses such as the coxsakie viruses (data not shown).

Table V summarizes the double antibody ELISA studies performed thus far. A selection of cases are shown in Figures 4–11. Table one demonstrates that all of the basic criteria required by the multiple antigen theory of autoimmunity are satisfied. Antibodies against VWF bind to antibodies against pgp1b (Figures 4 and 5) demonstrating that the known complementarity between the antigens is reflected in the antibodies they elicit. Antibodies against CMV bind to antibodies against GAS (Figure 6), demonstrating that these antibodies can be complementary (or idiotype–antiidiotype). GAS antibodies bind to VWF antibodies (Figure 7), demonstrating that GAS and VWF are complementary. CMV antibodies bind to pgp1b antibodies (Figure 8), demonstrating that these antibodies are also complementary. However, some CMV antibodies also bind to VWF antibodies (Figures 9 and 10), and some GAS antibodies also bind to pgp1b antibodies (Figure 11).

### Discussion

Homology searching reveals that both CMV and GAS contain multiple antigens that may mimic both VWF and platelet glycoprotein 1b within the regions of VWF and pgp1b that are associated with their mutual binding. Thus, homology results suggest that CMV and GAS may elicit complementary antibodies in

Table III. Von Willebrand factor (factor IX) sequence homologies with group A streptococcus proteins. The von Willebrand factor region used for homology searching is that associated with platelet glycoprotein binding (see text).

accordance with the criteria set forth by the complementary antigen theory of autoimmunity.

The results of double antibody ELISAs confirm the homology data predictions that CMV and GAS antigens are homologous to VWF and pgp1b antigens. VWF antibodies are complementary to platelet glycoprotein 1b antibodies as would be expected from the known complementarity of their antigenic sequences (Figures 4 and 5). CMV antibodies are complementary to GAS antibodies, again as predicted from the homology data and from theory (Figure 6). Again in accord with the homology results, there appear to be multiple sets of CMV antibodies with different specificities that are complementary to GAS antibodies. Some GAS antibodies are complementary to both VWF and pgp1b antibodies (Figures 7 and 11), and some CMV antibodies are complementary to both VWF and pgp1b (Figures 8–10). Thus, there are two possible ways in which CMV and GAS may interact. CMV antigens may mimic VWF antigens while GAS antigens mimic pgp1b; or CMV antigens may mimic pgp1b antigens while GAS antigens mimic VWF antigens; or both may occur simultaneously.

Unexpectedly, these results also suggest that GAS may contain a significant number of complementary antigens within itself that mimic both VWF and pgp1b so that it is capable of satisfying the complementary antigen theory of autoimmunity by itself. Similary for CMV, we have thus far, however, found no set of GAS antibodies that bind to each other, nor any set of CMV antibodies that are complementary, so the possibility of a complex agent inducing sets of complementary antibodies remains possible but conjectural.

In sum, our data satisfy the theoretical predictions made about possible induction of ITP by the complementary antigen theory of autoimmunity using antigenic sequences and antibodies induced by a pair of infectious agents both associated epidemiologically with

Table IV. Platelet glycoprotein 1b sequence homologies with cytomegalovirus proteins. The glycoprotein region used for homology searching is that associated with von Willebrand factor binding (see text).

risk for ITP. Several steps clearly remain to be satisfied before the theory can be considered validated, however, and these include demonstrations that the CMV and GAS antibodies identified here actually recognized ITP related antigens (VWF and platelet glycoproteins); that the CMV and GAS antigens are themselves complementary (i.e. bind to each other) as do the proteins they mimic (VWF and pgp1b); and most importantly that a combination of such antigens is, in fact, capable of inducing ITP experimentally in an animal model. Much

Table V. Summary of double antibody ELISA experiments used to test whether for possible idiotype–antiidiotype relationships between group A streptococcus antibodies, cytomegalovirus antibodies, platelet glycoprotein antibodies, and von Willebrand factor antibodies. See figures for examples of positive (+) results. Blanks indicate that the experiment was not done.

| Double antibody elisas | (1) Gt × GAS | (2) Rbt × GAS | (3) G. pig × CMV | (4) Gt × CMV | (11) Gt × VWF | (12) Shp × VWF | (13) Gt × Plt gp Ib | (14) MAB × Plt gp Ib |
|---|---|---|---|---|---|---|---|---|
| (1) Gt × GAS | | | + | | + | − | + | − |
| (2) Rbt × GAS | | | | − | − | − | − | |
| (3) G. pig × CMV | + | − | | − | − | + | | + |
| (4) Gt × CMV | | − | − | | − | − | − | − |
| (5) MAB × CMV IEA | − | − | | − | − | | | |
| (6) MAB × CMV EA | − | − | | − | − | + | − | |
| (7) MAB × CMV gpB | − | − | | − | − | + | − | |
| (8) MAB × CMV gp gH | − | − | | − | − | + | | |
| (9) MAB × CMV SLA | − | − | | − | − | + | − | |
| (10) MAB × CMV 65 kD | − | − | | − | − | + | | |
| (11) Gt × VWF | + | − | − | − | | | + | − |
| (12) Shp × VWF | − | − | + | − | | | + | + |
| (13) Gt × Plt gp Ib | + | − | | − | + | + | | |
| (14) MAB × Plt gp Ib | − | | + | − | − | + | | |
| (15) Shp × Plt gp IIb/IIIa | − | | + | − | − | | | + |
| (16) MAB × EBV | − | | | | | | | |
| (17) Gt × HIV − 1 | − | | | | − | − | − | − |
| (18) Gt × HSV-1 | − | | | | − | − | − | − |
| (19) Shp × HSV-2 | − | | | | − | − | − | − |
| (20) Gt × HBsAg | − | | | | − | − | − | − |
| (21) Rbt × *M. tuberculosis* | | − | | | − | − | − | − |

| Antibody | Manufacturer | Catalog No. |
|---|---|---|
| (1) Goat × Group A *Streptococcus*, HRP | Biodesign International, Kennebunkport, ME, USA | B65150P |
| (2) Rabbit × Group A *Streptococcus* | Biodesign International | B83601R |
| (3) Guinea pig × Cytomegalovirus | Biodesign International | B47821P |
| (4) Goat × Cytomegalovirus, virions, HRP | Biodesign International | B65273G |
| (5) MAB × Cytomegalovirus, pp72 immediate early antigen | Biodesign International | C8A022M |
| (6) MAB × Cytomegalovirus, 65 kDa early antigen | Biodesign International | C86314M |
| (7) MAB × Cytomegalovirus, Glycoprotein B | Biodesign International | C65826M |
| (8) MAB × Cytomegalovirus, Glycoprotein gH | Biodesign International | C65861M |
| (9) MAB × Cytomegalovirus, 45 kDa Structural late antigen | Biodesign International | C65879M |
| (10) MAB × Cytomegalovirus, 65 kD Late major matrix protein | Biodesign International | C65083M |
| (11) Goat × von Willebrand Factor, HRP | Cedarlane Laboratories LTD, Ontario, Canada | CL20175HP |
| (12) Sheep × von Willebrand Factor | Biodesign International | K90054C |
| (13) Goat × Platelet gp Ib | Santa Cruz Biotechnology, Inc., Santa Cruz, CA, USA | sc-7071 |
| (14) MAB × Platelet gp Ib | Biomeda Corporation, Foster City, CA, USA | V1103 |
| (15) Sheep × Platelet gp IIb/IIIa | Cedarlane Laboratories LTD | CL20069A |
| (16) MAB × Epstein-Barr virus | Biodesign International | C65221M |
| (17) Goat × HIV-1, HRP | Biodesign International | B65873G |
| (18) Goat × HSV-1, HRP | Biodesign International | B65134G |
| (19) Sheep × HSV-2, HRP | Biodesign International | B651245 |
| (20) Goat × Hepatitis B surface antigens, (HBsAg), (ad/ay), HRP | Biodesign International | B65804P |
| (21) Rabbit × *M. tuberculosis*, HRP | Biodesign International | B65601C |

work clearly remains to be done to test the theory that CMV and GAS may interact to produce ITP.

We also caution that the choice of this particular pair of infectious agents does not imply that other pairs of agents cannot be involved in the induction of ITP. ITP may have many causes, so that combinations of staphylococci with rubella or varicella, or helicobacter with some herpes virus might also be implicated in some forms of ITP. Indeed, there is no reason to think that ITP need have one cause, nor one mechanism.

### *Antisense peptides as complementary antigens*

Pendergraft et al. (2004) have recently described a human form of autoimmunity triggered by cPR-3 (105–201), a protein complementary to human autoantigen proteinase-3. Their discovery has significantly raised interest in the possibility that complementary antigens play a role in the induction of autoimmunity (Shoenfeld 2004, McGuire and Holmes 2005). There are, however, a number of ambiguities and technical difficulties associated with their work that need to be addressed in the context of the theory being proposed here.

To begin with, Pendergraft et al. (2004) have adopted a definition of complementarity that is significantly more limited than that employed here. The definition of complementarity employed here is that antigens must be capable of stereospecific binding to each other and that stereospecific binding must be manifested by the induction of pairs of complementary antibodies (or T-cells) that act like idiotype–antiidiotype pairs. Pendergraft et al. (2004, 2005), in

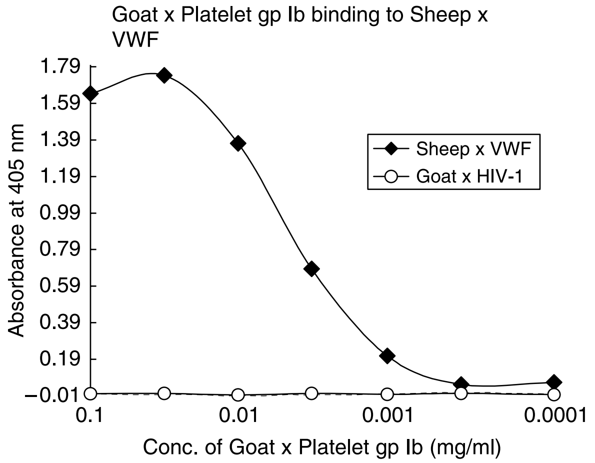

Figure 4. Double antibody ELISA.

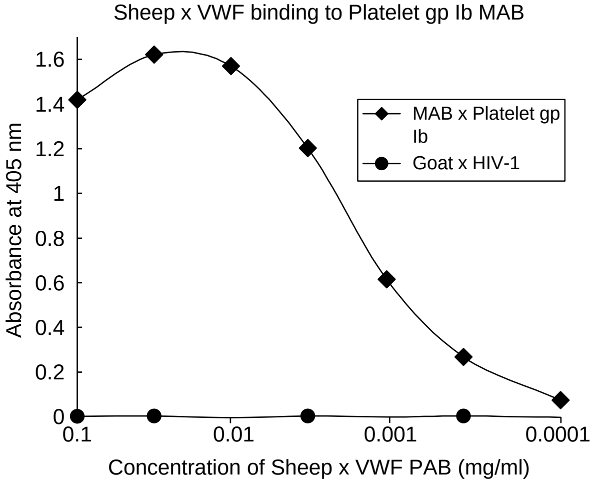

Figure 5. Double antibody ELISA.

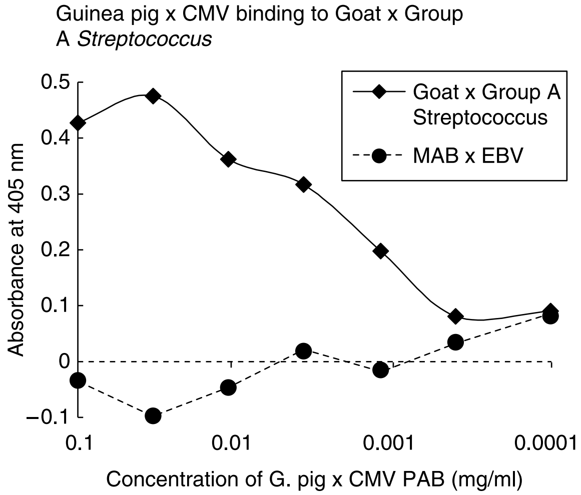

Figure 6. Double antibody ELISA.

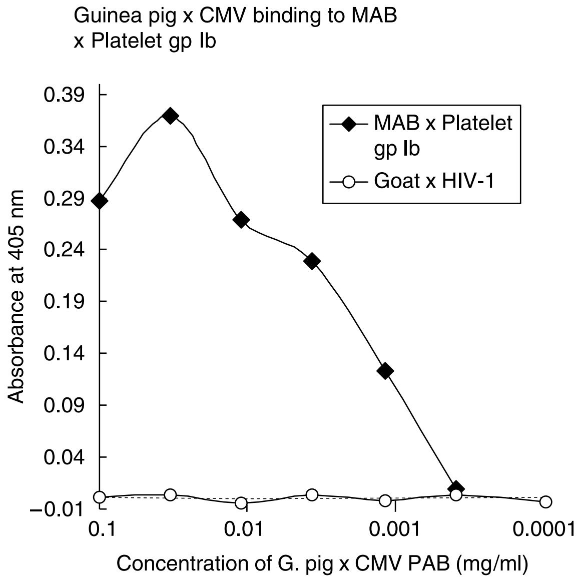

Figure 8. Double antibody ELISA.

contrast, have defined complementarity in terms of Blalock's concept of antisense proteins. In essence, Blalock has proposed that if each chain of double-stranded DNA could be translated into a protein, the resulting proteins would have an antisense relationship equivalent to the antisense relationship of the original DNA chains or their respective RNAs (Blalock and Smith 1984, Tropsha et al. 1992). These antisense proteins, or their peptide fragments, would bind to each other, just as the antisense strands of DNA or RNA bind to each other. The physicochemical basis of this protein–protein binding is hydropathic complementarity, where hydropathy is a complex measure of hydrophilicity/hydrophobicity, side chain size, etc. There are two major problems with applying antisense protein concepts to autoimmunity, one involving intrinsic problems with Blalock's concept, the other involving ambiguities in the way that the concept is being applied by Pendergraft et al. (2004, 2005).

The problem with Blalock's concept of antisense proteins is that there is almost no physicochemical data demonstrating that proteins derived from complementary chains of DNA are chemically complementary, while there are many physicochemical studies that show that such proteins do not bind to each other (reviewed in Root-Bernstein and Holsworth 1998, Siemion et al. 2004). There is little evidence that such antisense proteins induce complementary antibodies that act like idiotype–antiidiotype pairs and much that demonstrates failure to do so. And there is an alternative theory of antisense peptides, in which the complementary strands of

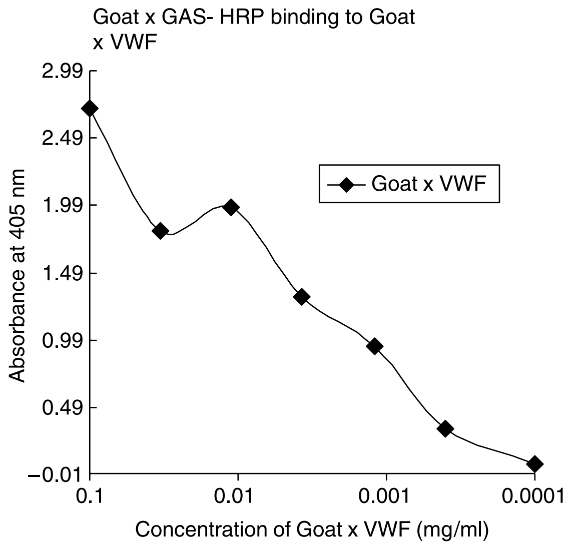

Figure 7. Double antibody ELISA.

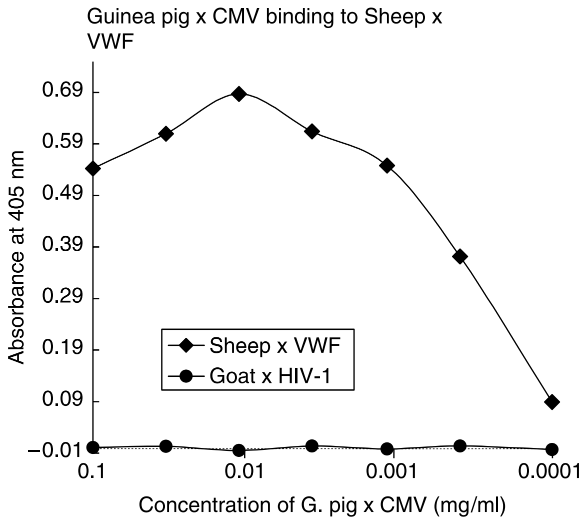

Figure 9. Double antibody ELISA.

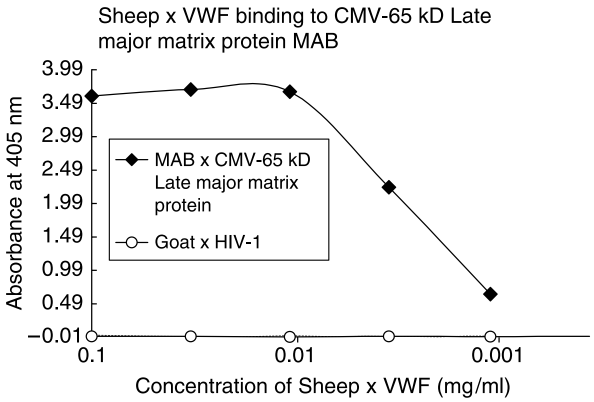

Figure 10. Double antibody ELISA.

DNA are read in parallel (that is, one chain is read "backwards" from the other) for which there is significant data demonstrating the production of complementary proteins, and which can explain most of the supposed data for Blalock's hypothesis (Root-Bernstein and Holsworth 1998, Siemion et al. 2004). Thus, it is not clear that Pendergraft's et al. (2004, 2005) demonstration that their proteins are "complementary" according to Blalock's criteria has any meaning in terms of whether the proteins would bind to each other or produce complementary antibodies. These are points that need to be experimentally demonstrated.

More importantly, none of the cases of antigen or antibody complementarity described in this paper satisfy the Blalock criteria. VWF and pgp1b do not appear to bind to each other according to hydropathic complementarity (data not shown). We have previously demonstrated that hydropathic complementarity cannot explain insulin–glucagon complementarity, nor the ability of these proteins to self-aggregate (Root-Bernstein 2005b). And there is

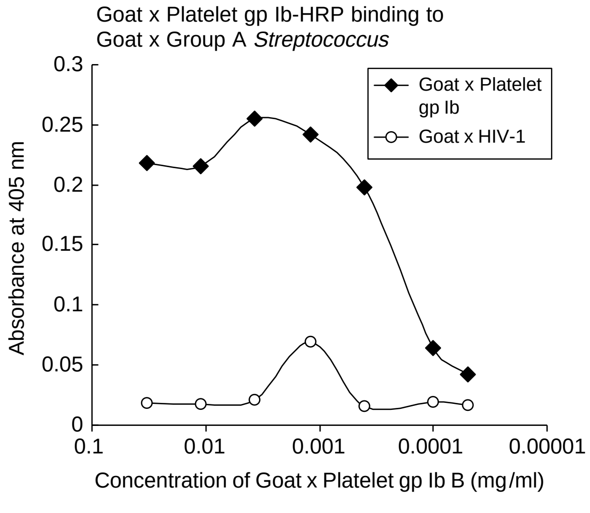

Figure 11. Double antibody ELISA.

clearly no application of the antisense concept to the interaction of myelin basic protein with MDP or other bacterial cell-wall derived adjuvants, which are primarily polysaccharide based. Thus, at the very least, the proposal that antisense peptides, hydropathic complementarity, or Blalock's concepts are at the root of autoimmune processes must be regarded as being of limited value.

There also appears to be some ambiguity as to how the concept of complementarity is being applied by Pendergraft et al. (2004, 2005), and by those writing about their findings. Pendergraft et al. (2004) write that, "The theory proposes that the inciting immunogen that elicits a cascade of immunological events is not the self-antigen (the autoantigen) or its mimic but rather a protein that is complementary..." This proposal seems to put emphasis on the complementary protein as the single agent necessary to induce autoimmunity and is not, therefore, equivalent to the theory of complementary antigens proposed here, which requires a pair of complementary antigens to break self-tolerance, Pendergraft's mechanism appears to be much more similar to Plotz's theory that antiidiotype antibodies are the cause of autoimmunity. In Plotz's (1983) theory, viral capsid proteins use host cellular receptors to infect cells. The host responds by making antibodies to the capsid proteins. These anti-capsid proteins mimic the binding specificity of the cellular receptors. If antiidiotype antibodies are produced to these anti-capsid antibodies, then these will mimic the specificities of the viral capsid proteins themselves, and attack the host cellular receptors. This scenario seems to be very similar to Pendergraft et al.'s theory that an antisense peptide will induce antibodies that in turn evoke antisense antibodies that become autoreactive.

On the other hand, McGuire and Holmes (2005) have clearly interpreted the Pendergraft et al. data in a manner that makes it much more similar to the theory proposed here. McGuire and Holmes argue that each protein generated by each strand of DNA will result in an antigen that induces an appropriate antibody. They assume that the antisense proteins encoded by antisense genes will be antigenically complementary and that the result will be antibodies that act like idiotype–antiidiotype pairs. This is a pretty story, but one for which even their references provide no substance. We are not, therefore, convinced that the application of antisense peptides and hydropathic complementarity to autoimmune research will do anything more than confuse thinking about how properly to apply the concept of molecular complementarity to immunology.

### *Rethinking Koch's postulates for autoimmune diseases*

In concluding, it should be noted that the complementary antigen theory of autoimmunity is incompatible with a strict reading of Koch's postulates for the identification of the cause of disease (Root-Bernstein 1991b). In Koch's model, diseases are caused by single agents. Disease causation can be established by: isolating in pure culture a single agent from a diseased organism; inoculating the pure agent into a healthy organism; observing the disease develop in the previously healthy organism; and reisolating the disease-associated agent from the now ill organism. The complementary antigen theory of autoimmunity predicts that Koch's postulates will not yield the cause of any autoimmune disease since no single antigen will be capable of inducing an autoimmune disease, and it will be a rare phenomenon for a single infectious agent to carry the requisite complementary set of antigens. A revised set of postulates are needed to test the complementary antigen theory: two or more purified agents must be associated with the autoimmune disease; none of the purified agents will able to induce the autoimmunity individually; autoimmunity will only be induced with a specific combination of the purified disease-associated agents. It is, of course, likely that none of the agents will be present by the time the autoimmunity is diagnosed, as the immune system may very well have eliminated the agents prior to targeting the tissues or organs of the organism itself. Thus, evidence of active immunity may need to replace isolation of the disease agents themselves in the determination of what agents are most likely correlated with any particular form of autoimmunity. In addition, it must be stressed that only specific pairs of purified agents (such as CMV with mycobacteria), both present simultaneously are predicted to be associated with autoimmunity; encountering one agent significantly prior to the other will lead to protection against autoimmunity. An equally important point to stress is that random sets of infections, such as most people encounter throughout their lives, are unlikely to contain the necessary complementary antigens and will not lead to autoimmunity. It is not the fact that combined agents are encountered by the immune system that leads to autoimmunity but the fact that a specific pair of agents that are related by molecular complementarity are encountered simultaneously by the immune system.

### Acknowledgements

We thank Maurine Bernstein for funding this research.

### References

Balint JP, Jr, Jones FR. 1994. Detection of elevated anti-idiotypic antibody levels in immune thrombocytopenic patients expressing antiplatelet antibody. Blood 84:664–669.

Bar Meir E, Amital H, Levy Y, Kneller A, Bar-Dayan Y, Shoenfeld Y. 2000. Mycoplasma-pneumoniae-induced thrombotic thrombocytopenic purpura. Acta Haematol 103:112–120.

Billingham MEJ, Carney S, Butler R, Colston MJ. 1990. A mycobacterial heat shock protein induces antigen-specific suppression of adjuvant arthritis, but is not itself arthritogenic. J Exp Med 171:339–344.

Blalock JE, Smith EM. 1984. Hydropathic anti-complementarity of amino acids based on the genetic code. Biochem Biophys Res Commun 121:203–207.

Candelli M, Nista EC, Pignataro G, Gasbarrini G, Gasbarrini A. 2003. Idiopathic thrombocytopenic purpura and *Helicobacter pylori* infection. Scand J Gastroenterol 38:569–576.

Clancy R, Trent R, Danis V, Davidson R. 1980. Autosensitization and immune complexes in chronic idiopathic thrombocytopenic purpura. Clin Exp Immunol 39:170–179.

Cohen IR, Miller A, editors. 1994. Autoimmune disease models. A Guidebook. New York: Academic Press.

Cruz MA, Diacovo TG, Emsley J, Liddington R, Handin RI. 2000. Mapping the glycoprotein Ib-binding site in the von Willebrand Factor A1 domain. J Biol Chem 275:19098–20017.

Emsley J, Cruz M, Handin R, Liddington R. 1998. Crystal structure of the von Willebrand Factor A1 domain and implications for the binding of platelet glycoprotein Ib. J Biol Chem 273: 10396–10410.

Esquivil PS, Rose NR, Kong YM. 1977. Induction of autoimmunity in good and poor responder mice with mouse thyroglobulin and lipopolysaccharide. J Exp Med 145:1250–1263.

Fisgin T, Yarali N, Duru F, Kara A. 2003. CMV-induced immune thrombocytopenia and excessive hematogones mimicking an acute B-precursor lymphoblastic leukemia. Leukemia Res 27:193–196.

Hamner DL, Lyon RM, Emans JB. 1996. Sudden death of a child who had pain in the knee and vericella. A case report. J. Bone Joint Surg 78A:594–596.

He R, Reid DM, Jones CE, Shulman NR. 1995. Extracellular epitopes of platelet glycoprotein Ibα reactive with serum antibodies from patients with chronic idiopathic thrombocytopenic purpura. Blood 86:3789–3799.

He R, Reid DM, Jones CE, Shulman NR. 1994. Spectrum of Ig classes, specificities, and titers of serum antiglycoproteins in chronic idiopathic thrombocytopenic purpura. Blood 83:1024–1037.

Hou M, Stockelberg D, Kutti J, Wadenvik H. 1997. Immunoglobulins targeting both GPIIb/IIIa and GPIb/IX in chronic idiopathic thrombocytopenic purpura (ITP): Evidence for at least two different IgG antibodies. Br J Haematol 98:64–73.

Huang X, Miller W. 1991. A time-efficient, linear-space local similarity algorithm. Adv Appl Math 12:337–349.

Humblot S, Martin T, Pasquali JL, Korganow AS. 2001. Blood coagulation disorders during primary Cytomegalovirus infection. Arch Intern Med 161:2149–2150.

Ichiche M, Fontaine C, Lacor P. 2003. Severe thromocytopenia secondary to cytomegalovirus infection in an immunocompetent adult. Eur J Intern Med 13:56–59.

Kahane S, Dvilansky A, Estok L, Nathan I, Zolotov Z, Sarov I. 1981. Detection of anti-platelet antibodies in patients with idiopathic thrombocytopenic purpura (ITP) and in patients with rubella and herpes group viral infections. Clin Exp Immunol 44:49–57.

Kouwabunpat D, Hoffman J, Adler R. 1999. Vericella complicated by group a streptococcal sepsis and osteonecrosis. Pediatrics 104:967–969.

Krook A, Soos MA, Kumar S, Siddle K, O'Rahilly S. 1996. Functional activation of mutant human insulin receptor by monoclonal antibody. Lancet 347:1586–1590.

Kurata Y, Hayashi S, Aochi H, Nagamine K, Oshida M, Mizutani H, Tomiyama Y, Tsubakio T, Yonezawa T, Tarui S. 1987. Analysis of antigen involved in circulating immune complexes in patients with idiopathic thrombocytopenicpurpura. Clin Exp Immunol 67:293–298.

Musaji A, Vanhoorelbeke K, Deckmyn H, Coutelier J-P. 2004. New model of transient strain-dependent autoimmune thrombocytopenia in mice immunized with rat platelets. Exp Hematol 32:87–91.

McGuire KL, Holmes DS. 2005. Role of complementary proteins in autoimmunity. Trends Immunol 26:367–372.

McMillan R. 2003. Antiplatelet antibodies in chronic adult immune thrombocytopenic purpura: Assays and epitopes. J Pediatr Hematol Oncol 25:S57.

Morrow WJW, Wharton M, Stricker RB, Levy JA. 1986. Circulating immune complexes in patients with acquired immune deficiency syndrome contain the AIDS-associated retrovirus. Clin Immunol Immunopahtol 40:515–524.

Morrow WJW, Isenberg DA, Sobol RE, Stricker RB, Kieber-Emmons T. 1991. AIDS virus infection and autoimmunity: A perspective of the clinical, immunological, and molecular origins of the autoallergic pathologies associated with HIV disease. Clin Immunol Immunopathol 58:163–174.

Nardi M, Karpatkin S. 2000. Antiidiotype antibody against platelet anti-GPIIIa contributes to the regulation of thromocytopenia in HIV-1-ITP patients. J Exp Med 191:2093–2100.

Oyaizu N, Yasumizu R, Miyama-Inaba M, Nomura S, Yoshida H, Miyawaki H, Shibata Y, Mitsuoka S, Yasunaga K, Morii S, Good RA, Ikehara S. 1988. (NZW × BXSB)$F_1$ Mouse: A new animal model of idiopathic thrombocytopenic purpura. J Exp Med 167:2017.

Pearson WR, Lipman DJ. 1988. Improved tools for biological sequence comparison. Proc Natl Acad Sci USA 85:2444.

Pendergraft, WF, 3rd, Preston GA, Shah RR, et al. 2004. Autoimmunity is triggered by cPR-3 (105–201), a protein complementary to human autoantigen proteinase-3. Nat Med 10:72–79.

Pendergraft WF, et al. 2005. Autoantigen complementarity: A new theory implicating complementary proteins as initiators of autoimmune disease. J Mol Med 83:12–25.

Plotz PH. 1983. Autoantibodies are anti-idiotype antibodies to antiviral antibodies. Lancet 2:824–826.

Prakken BJ, van der Zee R, Anderton SM, van Kooten PJS, Kuis W, van Eden W. 1997. Peptide-induced nasal tolerance for a mycobacterial heat shock protein 60 T cell epitope in rats suppressed both adjuvant arthritis and nonmicrobially induced experimental arthritis. Proc Natl Acad Sci USA 94:3284–3289.

Puram V, Giuliani D, Morse BS. 1984. Circulating immune complexes and platelet IgG in various diseases. Clin Exp Immunol 58:672–679.

Rahal JJ, Jr, MacMahon HE, Weinstein L. 1968. Thromocytopenia and symmetrical peripheral gangrene associated with staphylococcal and streptococcal bacteremia. Ann Intern Med 69:35–43.

Reddy MM, Grieco MH. 1990. Elevated levels of circulating immune complexes in human immunodeficiency virus infection. J Clin Lab Anal. 4:95–98.

Root-Bernstein RS, Westall FC. 1983. Sleep factors: Do muramyl peptides activate the serotonin binding sites? Lancet i :653.

Root-Bernstein RS, Yurochko F, Westall FC. 1986. Clinical suppression of experimental allergic encephalomyelitis by muramyl dipeptide "adjuvant". Brain Res Bull 17:473476.

Root-Bernstein RS, Westall FC. 1986. Complementarity between antigen and adjuvant in the induction of autoimmune diseases a dual antigen hypothesis. J Infer Deduct Biol 2:137.

Root-Bernstein RS, Westall FC. 1990. Serotonin binding sites. II. Muramyl dipeptides bind to serotonin binding sites on myelin basic protein, LHRH, MSH, and ACTH. Brain Res Bull 25:827841.

Root-Bernstein RS. 1991a. Multiple antigen mediated autoimmunity (MAMA) in AIDS: A possible model for post infectious autoimmunity. Res Immunol 141:321–339.

Root-Bernstein RS. 1991b. Self, nonself, and the paradoxes of autoimmunity. In: Tauber AI, editor. Organism and the development of self. Boston: Kluwer. p 159209.

Root-Bernstein RS, Hobbs SH. 1992. Homologies between mycoplasma adhesion peptide, CD4, and class II MHC proteins: A possible mechanism for HIVmycoplasma synergism in AIDS. Res Immunol 142:5.

Root-Bernstein RS, Hobbs SH. 1993. Does HIV "piggyback" on viruses, bacteria, and sperm? Implications for transmission, synergism, and autoimmunity in AIDS. J Theor Biol 160:249–264.

Root-Bernstein RS, Dewitt SH. 1994. CD4 similarity to proteins of infectious agents associated with AIDS and their role in autoimmunity. Med Hypoth 43:361–371.

Root-Bernstein RS. 1995. Preliminary evidence of idiotype antiidiotype immune complexes crossreactive with lymphocyte antigens in AIDS and lupus. Med Hypoth 44:20–27.

Root-Bernstein RS, Dewitt SH. 1995. Semen alloantigens as possible inducers of lymphocytotoxic autoimmunity in AIDS and ICL. Genetica 95:133–156.

Root-Bernstein RS, Holsworth DD. 1998. Antisense peptides: A critical review. J Theor Biol 190:107–119.

Root-Bernstein RS, Dobbelstein C. 2001. Insulin binds to glucagon forming a complex that is hyper-antigenic and inducing complementary antibodies having an idiotype–antiidiotype relationship. Autoimmunity 33:153–169.

Root-Bernstein RS. 2004. Antigenic complementarity among AIDS-associated infectious agents and molecular mimicry of lymphocyte proteins as inducers of lmphocytotoxic antibodies and circulating immune complexes. J Clin Virol 31S:S16–S25.

Root-Bernstein RS, Rallo A. 2004. Antigenic complementarity resulting in idiotype–antiidiotype immune complexes: Possible contributor to AIDS pathogenesis and autoimmunity. Autoimmunity 37:203–210.

Root-Bernstein RS. 2005a. Antigenic complementarity between HIV and other AIDS-associated infections results in idiotype–antiidiotype antibody coplexes that cross react with lymphocyte proteins. Vaccine 23:2160–2163.

Root-Bernstein RS. 2005b. Peptide self-aggregation and peptide complementarity as bases for the evolution of peptide receptors: A review. J Mol Recognit 18:40–49.

Sakata H, Ikegami K, Nagaya K, Shirai M, Maruyama S. 1999. Thrombocytopenia caused by acquired cytomegalovirus infection in children. Pediatr Intern 41:113–114.

Shoelson SE, Marshal S, Horikoshi H, Kolterman OG, Rubenstein AH, Olefsky JM. 1986. Anti-insulin receptor antibodies in insulin-dependent diabetics may arise as autoantiidiotypes. J Clin Endocrinol Metab 63:56–61.

Shoenfeld Y. 2004. The idiotypic network in autommunity. Nat Med 10:17–18.

Shimizu A, Matsushita T, Kondo T, Inden Y, Kojima T, Saito H, Hirai M. 2004. Identification of the amino acid residues of the platelet glycoprotein Ib (GPIb) essential for the von Willebrand Factor binding by clustered charged-to-alanine scanning mutagenesis. J Biol Chem 279:16285.

Siemion IZ, Cebrat M, Kluczyk A. 2004. The problem of amino acid complementarity and antisense peptides. Curr Protein Pept Sci 5:507–527.

Silvestris F, DiLoreto M, Romito A, Grizzuti MA, Dammacco F. 1994. Distribution and antigenic analysis of circulating F(ab')2-reactive IgG in patients with HIV-1 infection. Clin Immunol Immunopathol 73:229–234.

Sonnabend JA. 1989. AIDS: An explanation for its occurrence among homosexual men. In: Ma P, Armstrong D, editors. The Acquired Immune Deficiency Syndrome and Infections of Homosexual Men. New York: Yorke Medical Books. p 449–469.

Stanojevic M, Zerjav S, Jevtovic D, Markovic L. 1996. Antigen/antibody content of circulating immune complexes in HIV-infected patients. Biomed Pharmacother 50:488–493.

Steinberg CM, Lefkovits I, editors. 1981. The Immune System. Basel & New York: Karger.

Stéphan F, Cheffi MA, Kaplan C, Maillet J-M, Novara A, Fagon J-Y, Bonnet F. 2000. Autoantibodies against platelet glycoproteins in critically ill patients with thrombocytopenia. Am J Med 108:554–562.

Takeuchi Y, Root-Bernstein RS, Shih JC. 1990. Peptide displacement of [3H] 5hydroxytryptamine binding to cortical membranes. Brain Res Bull 25:817–820.

Takahashi T, Guiro T, Shinohara K, Inoue Y, Sato Y, Fujii Y, Okubo M, Zaitsu Y, Ariyoshi K, Nakamura Y, Nawata R, Oka Y, Shirai M, Tanizawa Y. 2004. Molecular mimicry by *Helicobacter pylori* CagA protein may be involved in the pathogenesis of H. Pylori-associated chronic idiopathic thrombocytopenic purpura. Br J Haematol 124:91–95.

Titani K, Takio K, Handa M, Ruggeri ZM. 1987. Amino acid sequence of the von Willebrand factor-binding domain of platelet membrane glycoprotein Ib. Proc Natl Acad Sci USA 84:5610–5614.

Trent RJ, Clancy RL, Danis V, Basten A. 1980. Immune complexes in thrombocytopenic patients: Cause or effect? Br J Haematol 44:645–647.

Tropsha A, Kizer JS, Chiken IM. 1992. Making sense from antisense: A review of experimental data and developing ideas on sense-antisense peptide recognition. J Mol Recognit 5:43–54.

Ulmansky R, Cohen CJ, Szafer F, Moallem E, Fridlender ZG, Kashi Y, Naparestek Y. 2002. Resistance to adjuvant arthritis is due to protective antibodies against heat shock protein surface epitopes and the induction of IL-10 secretion. J Immunol 168:6463–6469.

Van Spronsen DJ, Breed WPM. 1996. Cytomegalovirus-induced thromocytopenia and haemolysis in an immunocompetent adult. Br J Haematol 92:219–220.

Vicente V, Kostel PJ, Ruggeri ZM. 1988. Isolation and functional characterization of the von Willebrand Factor-binding domain located between residues $His^1$–$Arg^{293}$ of the α-chain of glycoprotein Ib. J Biol Chem 263:18473–18482.

Wadenvik H, Stockelberg D, Hou M. 1998. Platelet glycoproteins as autoantibody targets in idiopathic thrombocytopenic purpura. Acta Paediatr Suppl 424:26–35.

Westall FC, Root-Bernstein RS. 1983a. An explanation of prevention and suppression of experimental allergic encephalomyelitis. Mol Immunol 20:169–177.

Westall FC, Root-Bernstein RS. 1983b. Optic neuritis—the mechanism of induction by dual antigen complex. Arch Opthalmol 101:486.

Westall FC, Root-Bernstein RS. 1986. The cause and prevention of postinfectious and postvaccinal encephalopathies in light of a new theory of autoimmunity. Lancet ii :251252.

Wright JF, Blanchette VS, Wang H, Arya H, Petric M, Semple JW, Chia WK, Freedman J, Chia, Freedman J. 1996. Characterization of platelet-reactive antibodies in children with varicella-associated acute immune thrombocytopenic purpura (ITP). Br J Haematol 95:145.

Yang X-D, Gasser J, Feige U. 1992. Prevention of adjuvant arthritis in rats by a nonapeptide from the 65-kD mycobacterial heat shock protein: Specificity and mechanism. Clin Exp Immunol 87:99–104.

Zandman-Goddard G, Shoenfeld Y. 2002. HIV and autoimmunity. Autoimmun Rev 1:329–337.

Zhang ZY, Lee CS, Lider O, Weiner HL. 1990. Suppression of adjuvant arthritis in Lewis rats by oral administration of type II collagen. J Immunol 145:2489–2493.

